# Knowledge, practices and attitudes on antibiotics use in Cameroon: Self-medication and prescription survey among children, adolescents and adults in private pharmacies

**DOI:** 10.1371/journal.pone.0212875

**Published:** 2019-02-28

**Authors:** Grace-Ange Elong Ekambi, Cécile Okalla Ebongue, Ida Calixte Penda, Emmanuel Nnanga Nga, Emmanuel Mpondo Mpondo, Carole Else Eboumbou Moukoko

**Affiliations:** 1 Pharmaceutical Sciences Department, Faculty of Medicine and Pharmaceutical Sciences, University of Douala, Douala, Cameroon; 2 Biological Sciences Department, Faculty of Medicine and Pharmaceutical Sciences, University of Douala, Douala, Cameroon; 3 Clinical Sciences Department, Faculty of Medicine and Pharmaceutical Sciences, University of Douala, Douala, Cameroon; 4 Centre Pasteur Cameroon, Yaoundé, Cameroon; University of Campania, ITALY

## Abstract

Benefits of antibiotics are threatened by the self-medication, people’s lack of knowledge and inappropriate use of antibiotics, especially in developing countries. This study was designed to determine knowledge; attitudes and practices toward antibiotics use in an urban community, and evaluate the factors that are associated with antibiotic use. Between January and March 2015, a cross sectional and prospective study was conducted in all pharmacies within the Douala IV health district, Cameroon. Anonymous interviews including both open and closed ended questions were conducted in participants selected by convenience sampling Descriptive and logistic regression analysis were performed using StataSE11 software (version 11 SE) and R software (version 3.1.1) in data analysis. Overall 402 (33.7%) of 1,192 customers purchased antibiotics and of these, 47% bought antibiotics without a prescription. 60.7% of purchased antibiotics was for adult ‘patients and around 60% of parents carried out self-medication on their children. The vast majority reported that all microbes can be treated with antibiotics (88.3%). The belief that antibiotics are appropriate for bacterial infections was more common among those with a higher level education (OR = 4.03, 95%CI:1.89–8.57, p<0.0001) and among public/private servants (OR = 2.47, 95%CI:1.21–5.08, p = 0.013). Physicians provide less explanations about antibiotics are and their potential side effects than the pharmacy auxiliaries (OR = 0.205, 95%CI = 0.09–0.46, p<0.0001), but more than pharmacists (OR = 3.692, 95%CI:1.44–9.25, p = 0.005). Indications on antibiotics use were 7 times more given to customers with a prescription compared to those without a prescription (OR = 7.37, 95% CI = 2.13–25.43, p = 0.002). Adult male (OR = 2.32, 95%CI:1.24–4.34, p = 0.009) and higher education (OR = 2.05, 95%CI:1.08–3.89, p = 0.027) were significantly associated with self-medication. Misuse, little "practical knowledge" and high self-medication confirm the unsatisfactory prescription and dispensing practices of the antibiotics in our country. These results highlight the important of the development and implementation appropriate guidelines for the responsible use of antibiotics for health care providers and health education targeting community members themselves.

## Introduction

Antibiotics have played a major role since the 20th century on reducing the morbidity and mortality associated with common infectious diseases and have therefore had an important impact on health care and human longevity [1,2]. Antibiotics and other antimicrobial agents are invaluable life savers, particularly in resource-limited countries where bacterial infections are predominant in both adults and childhood illness, contributing to the sustainable use of antibiotics which is the most important drugs of choice in the therapeutic arsenal [3].

This therapeutic and societal benefit of antibiotics appears to be threatened by abuse, self-medication, and misuse of antibiotics. In many countries, taking medicines, such as antibiotics, without a prescription has been a commonplace occurrence of everyday of life for many years. Younger children are often given medications by their parents, whereas older adolescents and adults may self-medicate themselves. The self-medication has always been favored by usually over-the-counter (OTC) drugs available in pharmacies and in local retail outlets. In the developed countries, 3–68% of antibiotics are sold without prescription [1,4–6] with a pediatric self-medication rates above 80% [7–9]. In developing countries, and particularly in Africa, studies have revealed a higher burden (30–85%) of self-medication [6,9–15], and in additions, the medications taken are often misused and the patients have wrong habits [1,2,16–18]. The self-medication of antibiotics remains a global problem and the misuse and overuse of antibiotics are further complicated by the spread of infections involving multi-drug resistant bacteria (MDRBs) which limit the action of drugs previously considered to be highly effective, as well as the shortage of novel antibiotics. The result is the risk of increasingly frequent therapeutic stalemates [2, 11, 19– 22]. Thus, tackling the global spread of antibiotic resistance is a high priority for the World Health Organization (WHO) which recommended creating increased awareness about self-medication and its control [23]. In first World Report on World Resistance, presented in 2014 in Geneva and covering 114 countries, the WHO warned “Unless we take significant actions to improve efforts to prevent infections and also change how we produce, prescribe and use antibiotics, the world will lose more and more of these global public health goods and the implications will be devastating” [1]. If the effective use of antibiotics is lost, common infections presently managed easily would re-emerge as potentially life-threatening.

Field experience suggests that the prescription and utilization of antibiotics are often suboptimal in Cameroon as well as many countries. In addition, antibiotics resistance is frequently identified and reported in Cameroon, and resistance to amoxicillin (55%) and cotrimoxazole (more than 65%) are the most frequently identified [24]. To date, many studies already have been undertaken to assess knowledge, attitudes and practices towards antibiotic use, the prevalence of self-medication and antibiotics prescribing in Africa [8, 9–15, 25, 26], but few evaluate the factors which could influence the prescription and self-medication of antibiotics among non-health care professionals in Africa [9]. These studies pointed out that the mothers with a higher score for knowledge of the antibiotics use and their hazards were less likely to give their children antibiotics without a prescription. In other countries, the antibiotics use without a prescription may result from several factors such as, having under 40 years of age, having attended a physician in the last 12 months, having an elementary education level, knowledge of antibiotic use, the patients’ experience with antibiotics [5, 27, 28], and the parents who did not use the Community Based Pediatrician as the sole source of information about drugs, or the misconception of antibiotics' effectiveness would be willing to self-medicate their children [29,30].

We have been unable to identify in the literature any other population based studies of knowledge, attitudes and practices towards antibiotic use, antibiotic prescribing, and factors that are associated with antibiotic use within the literature in the Cameroonian. Within in this context, this study was designed to use data from interviews and prescription records from private pharmacies in Douala (Littoral Region) to i) determine knowledge, attitudes and practices towards antibiotic use, ii) estimate the prevalence of self-medication and antibiotics prescribing, and iii) evaluate the factors that are associated with antibiotic use in Cameroon.

## Materials and methods

### i) Study design and population

A cross-sectional study was conducted for three months among individuals, and presenting to the 13 private pharmacies located at the Douala 4^th^ district in Cameroon. With an estimated average annual population growth rate of 5% the last 30 years and a current population of approximately 2,500,000 inhabitants (officially 1,931,977), Douala is therefore the largest city in Cameroon with an estimated annual average population growth rate of 5% the last 30 years. At the institutional level, Douala is organized in two levels, an urban community and six continental and insular districts. Located at the western entrance of Douala, the 4^th^ District is a highly cosmopolitan with people from diverse cultural backgrounds. Six of the 13 pharmacies were excluded from the study: 2 for low customer flow (3–5 people per day) and 4 for the repeated absence of the pharmacist who was to give the authorization for participation in the research study.

In the absence of reliable national and uncertainty data on the prevalence of people consuming antibiotics in Cameroon, we used data sources from the WHO [1] and assuming a prevalence of antibiotic use of 50%, it was determined that a minimal sample size of 390 would be needed and calculated using the Cochran’s formula [31]. We used a convenience sampling a non-probability sampling applicable in the study when the members of the population are convenient to sample. For limit the selection and information biases, participants were enrolled consecutively and participation in the study was voluntary.

The survey’s target population was all customers aged 15 years and over who visited the pharmacy and requested the purchase of antibiotics, with or without a prescription. The customers were asked verbally when they came to the pharmacy whether they would like to buy prescribed antibiotic or antibiotic for self-medication. If the answer was “no”, they will be excluded. If yes, they will be approached to participate in the study.

Administered questionnaire was conducted following a 1-week pre-test among 15 peoples to assess: i) understanding and acceptability of participants to the study and ii) to standardize and homogenize data collection in all pharmacies. Interview questions were formulated so as not to influence participants in their answers. After the pre-test, the modifications have been made on the knowledge of antibiotics: what is an antibiotic and what is its role.

The questionnaire was administered independently the same day by two persons (a 7-year pharmaceutical student-interviewer-1 and a 4^th^ year pharmaceutical student-interviewer-2) interviewing the customers within 5mn, about their purchase of drugs in the pharmacies to estimate inter-interviewer reproducibility. Customers did not receive any information sheets on antibiotics during these two interviews to minimize the risk of answer change.

Information sheets on antibiotics were provided to all customers after the interview and completing the questionnaire, with the emphasis on the importance of an antibiogram, a tool in infection control that can promote better self-management. This sheet allowed to have information on what are the antibiotics? what is antibiotic resistance? what is antibiogram? what is "inappropriate" use of antibiotics and what can physicians and other health professionals and the public do to fight gain the abuse of antibiotic consumption and antibiotic resistance.

This study was conducted in accordance with ethics directives related to research on humans in Cameroon. The Institutional Review Board of the University of Douala (IRB/UD) approved the study. Administrative authorization from each pharmacy was also obtained. Before enrollment and the administration of questionnaire, subjects were informed on the purpose and process of the investigation (background, goals, methodology, study constraints, data confidentiality, and rights to opt out from the study), and a signed informed consent was obtained from all participants who agreed to participate in the study in accordance with the Helsinki Declaration. Participation was voluntary, anonymous and without compensation.

### ii) Study questionnaire

They were open-ended (OEIQ) and closed-ended (CEIQ) interview questions, including a single answer, and multiple choices questions. Data collection sheets were used to collect data on socio-demographic characteristics (age, gender, place and year of birth, education and occupation and family and socio-economic background). In the second part of data, we sought to evaluate, knowledge and attitudes towards antibiotic use (definition of antibiotic and their role, knowledge about side effects/acuteness and dangers involved with the misuse of antibiotics, and be able to name them). The third part focused on self-medication with antibiotics or on antibiotics prescribing (medical reasons for antibiotics use, status of the prescriber or the counselor, why they self-medicate, who dispensed the antibiotics in the pharmacies, the person who had taken the antiobiotic, and which parent was most often involved with self-medication of minors types of antibiotics requested, medication) …

### iii) Statistical analysis

Categorical variables were expressed as frequencies, whereas numerical variables (Age) were presented as means and Standard deviation (SD), as data is normally distribution. Comparing proportions, we used Chi-square test or Fisher’exact test. Numerical values were compared using a parametric test, the Student-test. Only the variables significant in the univariate model were analyzed in a multivariate logistic regression model and taking into account all confounding factors (age, gender, education level, Marital status and occupation). All statistical analyzes were performed using Stata software (version 11 SE) and R software (version 3.1.1). Only p-values <0.05 were considered significant in all analyses.

## Results

### Characteristics of participants in population survey

**Fig 1:**
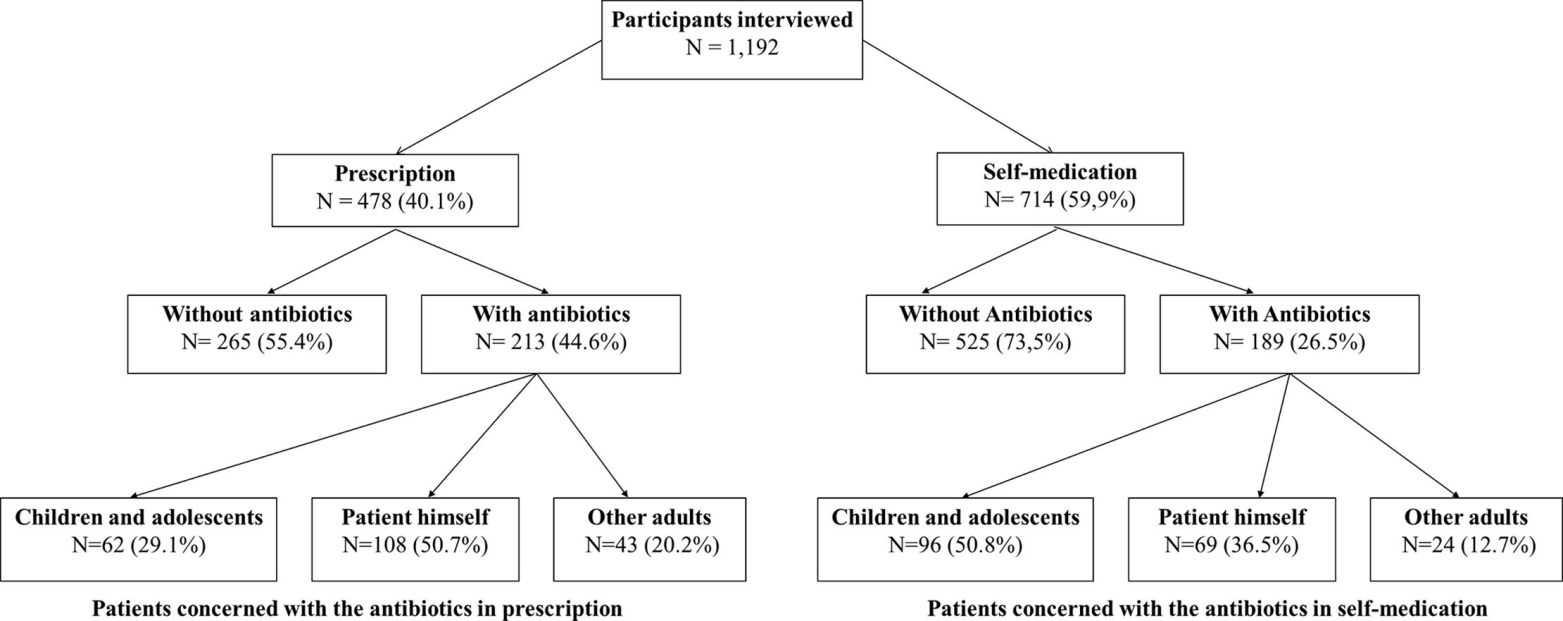
Flow chart of participants through the study according to the type of medication.

A total of 1,192 customers had been asked verbally when they came to the pharmacy whether they would like to buy prescribed antibiotic or antibiotic for self-medication in the seven selected pharmacies over a 3-months period in 2015, Slightly over a third (402, 34.7%) purchased antibiotics, and almost half (189, 47.%) of these customers purchased antibiotics without a prescription (Fig 1). All customers who purchased antibiotics agreed to participate.

Most antibiotics (244/402, 60.7%) were purchased for adults aged 15 years and over, and the remaining 39.30% (158/402) were purchased for children and adolescents. Around 72% of adults (177/244) had bought antibiotics for themselves and the remaining 27.46% had bought the medications for a third party. Overall, the male/female sex ratio amongst all participants who purchased antibiotics was 1.1/1 (209/193), and the mean age of the men was significantly higher compared to that of the female (36.75years *vs* 33.1years old; p = 0.0001) (Table 1).

**Table 1:** Characteristics by gender of participants visiting the pharmacies to purchase antibiotics.

	*Female* *N = 193*	*Male* *N = 209*	*Total* *N = 402*	^*p*^
**Age mean (SD),years**	33.1 (9.8)	36.76 (10.9)	35.02 (10.5)	0.0001
**School-education**				
Out-of-school	0 (0.0)	6 (2.9)	6 (1.5)	Reference
Primary	23 (11.9)	23 (11.0)	46 (11.4)	0.023
Secondary	105 (54.4)	111 (53.1)	216 (53.7)	0.020
University	65 (33.7)	69 (33.0)	134 (33.3)	0.021
**Marital status**				
Unmarried	61 (31.6)	62 (29.6)	123 (30.6)	Reference
Widowed/Divorced	8 (4.1)	1 (0.5)	9 (2.2)	0,023
Married/Concubinage	124 (64.3)	146 (69.9)	270 (67.2)	0,285
**Occupation**				
Pupils/Students	31 (16.1)	19 (9.1)	50 (12.4)	Reference
Unemployed	33 (17.1)	6 (2.9)	39 (9.7)	0,031
Public sector employee	13 (6.7)	18 (8.6)	31 (7.7)	0,108
Private sector employee	53 (27.5)	104 (49.8)	157 (39.1)	0,001
Informal sector	63 (32.6)	62 (29.7)	125 (31.1)	0,182

Data are number and/or proportion (%), unless otherwise indicated.

Overall, no difference was observed for educational level between men and women (p = 0.122) in our study population, but a significant difference was observed when comparing the different sub-groups of person with school-education and the person out-of-school (Table 1). Most of people who come to the pharmacy for the purchase of antibiotics are married or have a concubine and there is a statistical difference in the marital status by gender (p = 0.034). Overall, there is a significant difference in the distribution of occupational activity between men and women (p<0.0001). The majority of women have an informal activity (32.6% *vs* 29.7% for men) or remain unemployed (17.1% *vs* 2.4% of men) whereas, men had a stable activity in the private sector (49.8% *vs* 27.5% for women) and the frequency of this distribution was significantly different (p<0.0001).

### Keywords used to describe antibiotics, practical knowledge and perceptions of antibiotics

According to this survey, most (94.2%) of the respondents ≥15 years old reported that they know the role of antibiotics (Table 2). However, in response to the OEIQ “What is the role of antibiotics?”, those who chose “yes” gave rise to 9 categories and only 43.8% (165/377) stated that it is appropriate for bacterial infections.

**Table 2:** Identification of Customers’ perceptions of antibiotic use*.

	Customers’ population			p
	Female (N = 192)	Male (N = 208)	Total (N = 400)	
**Do you know antibiotics definition and their role**				
***Yes***	184 (95.8)	193 (92,8)	377 (94.2)	0.137
Treat all	14 (7.6)	12 (6.2)	26 (6.9)	0.371
Decrease fever	10 (5.4)	14 (7,2)	24 (6.4)	0,305
Treat microbes	166 (90.2)	167 (86.5)	333 (88.3)	0.170
Treat bacterial disease	81 (44.0)	84 (43.5)	165 (43.7)	0,502
Treat viral disease	25 (13.5)	20 (10.3)	45 (11.8)	0.210
Treat parasitic disease	11 (6,0)	12 (6.2)	23 (6.1)	0.547
Treat fungal disease	15 (8.1)	15 (7.8)	30 (7.9)	0.521
Calm pain	17 (9.2)	23 (11.9)	40 (10.6)	0.250
Fight against tiredness	1 (0.5)	7 (3.6)	8 (2.1)	0.039
**Do you know if antibiotics have side effects/acuteness**				
***Yes***	192 (100)	208 (100)	400 (100)	/
***Able to name some side effects***	120 (62.5)	113 (54.3)	233 (58.2)	0.060
Digestive disorders	35 (29.2)	33 (29.2)	68 (29.2)	0.555
Allergies/Itching	59 (49.2)	64 (56.6)	123 (52.8)	0.156
Dizziness	29 (24.2)	19 (16.8)	48 (20.6)	0.110
Tiredness	26 (21.7)	13 (11.5)	39 (16.7)	0.028
Other^$^	13 (10.8)	12 (10.6)	25 (10.7)	0.564

Data are number and/or proportion (%)

*, Under-15 years old were excluded from the analysis

^$^, Anorexia, headache, hum, deficient immune system, difficulty swallowing and, palpitations.

Public and private sector employees accounted for 60% (99/165) of respondents who correctly identified the role of antibiotics as treating bacterial infections (OR = 2.47, 95%CI:1.21–5.08, p = 0.013). Even more impressive, customers with a high school education level accounted for 94.5% (156/165) of correct responders (OR = 4.03, 95%CI:1.89–8.57, p<0.0001). Most of responders (88.33%) reported that antibiotic act on all microbes and 11.94% believed antibiotics also fight infections caused by viruses, and a non-negligible group (10.61%) also attested that antibiotics can be used to treat the pain.

Overall, respondents believe that abuse of antibiotics can lead to side effects, but just over half (58.2%) were able to name any side effects related to the consumption of antibiotics (Table 2). Participants named the side effects of allergy (52.8%), digestive disorders (29.2%) and dizziness (20.6%) most frequently. Women reported twice more the fatigue as side effects, while men reported more allergies/itching (p = 0.028). Twice more public/private sector employees (OR = 2.40, 95%CI:1.26–4.57, p = 0.008), and respondents with high school-education level (OR = 1.85, 95%CI: 1.20–2.86, p = 0.06) had a higher knowledge of side effects.

A small number (4%) of the interviewed adults believed that antibiotics can be used without medical advice and 86.65% agreed that they are dangerous.

### Antibiotics prescribing and reasons of antibiotics self-medication

**Fig 2:**
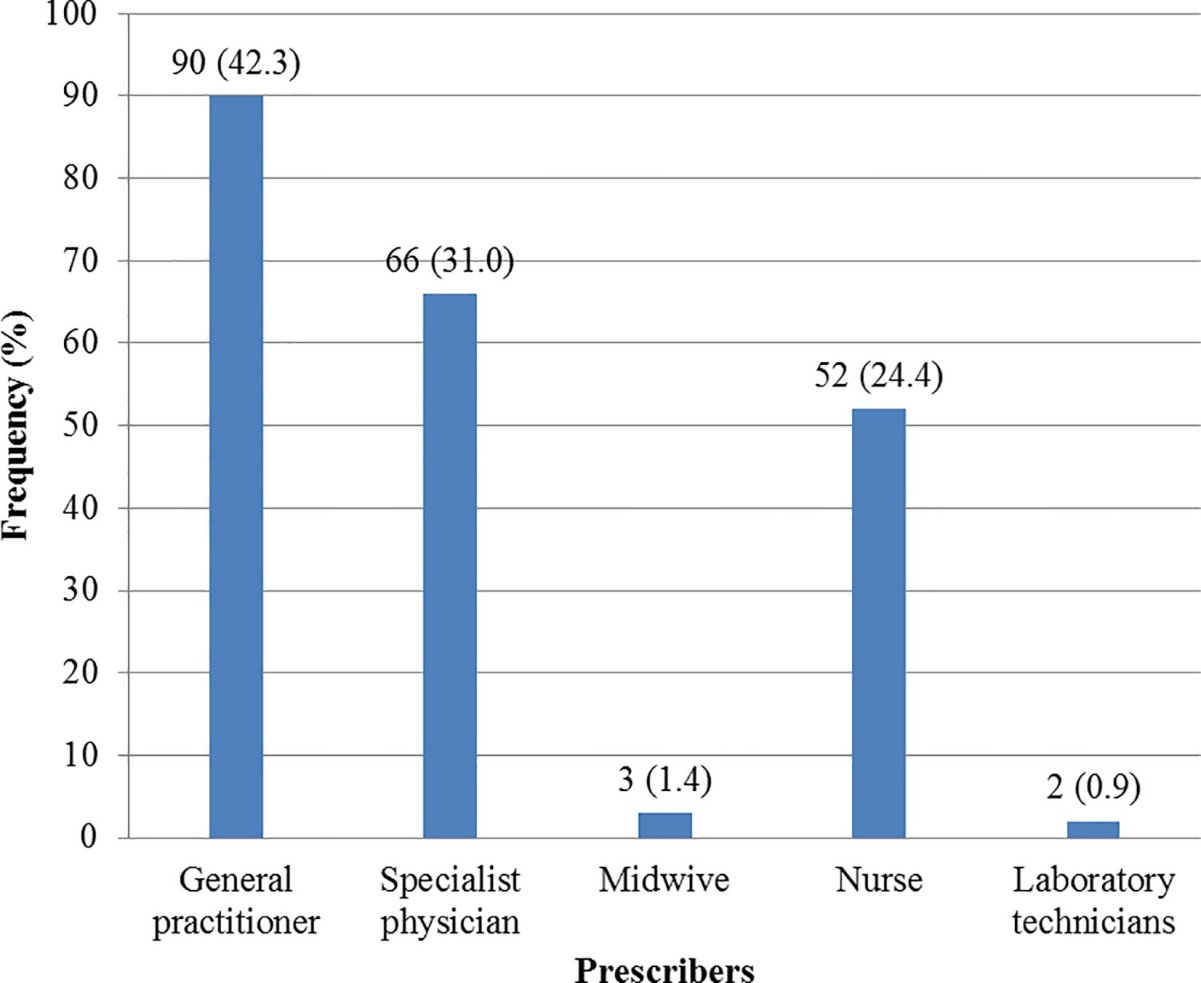
Antibiotics prescribers to outpatient in the Douala IV health district in Douala.

About 73.2% of antibiotics were prescribed by a practitioner (generalist or specialist) with 31.0% of antibiotic prescriptions issued by a specialist (Fig 2).

About one quarter (24.4%) of prescriptions were issued by nurses, while only 2.3% were issued by laboratory technicians and midwives. Only 2% of prescriptions had antibiogram results.

Overall, 93 (49.2%) and 58 (30.7%) respondents shared that they self-mediated after advice from healthcare worker, or by personal motivation (Table 3). Only 13.2% self-medicated with antibiotics due to the remoteness of healthcare facilities, and there was a trend for this reason to be more common among women more than men (p = 0.064). The main personal motivating for antibiotic self-medication was the recidivism/recurrent of disease symptoms that had been treated before and for which the person had already received a prescription (33.9%), drug taking practice (25.4%) or renewal previous prescription (23.7%). In multivariate analysis, only purchase advice by a third person (OR = 4.99, 95%CI:1.1–22.8, p = 0.038) and personal motivation (OR = 2.64, 95%CI:1.01–6.92, p = 0.048) were associated with self-medication compared to the difficulty accessing to health care among women.

**Table 3:** Sources of self-medication among patients concerned with the antibiotics and reasons for the antibiotics self-medication.

	Female (N = 83)	Male (N = 106)	Total (N = 189)	OR (95% CI)	^p^
Difficulties accessing healthcare	15 (18.1)	10 (9.43)	25 (13.2)	/	Reference
**Advice from healthcare worke**r	44 (53,0)	49 (46,2)	93 (49.2)	1.67 (0.68–4.1)	0.263
Pharmacist	20 (45.4)	17 (35.4)	37 (40.2)		
Practitioner	6 (13.6)	6 (12.5)	12 (13.0)		
Auxiliary pharmacy	11 (25.0)	15(31.2)	26 (28.3)		
Nurse	7 (15.9)	10 (20.8)	17 (18.5)		
**Advice from a third person**	3 (3.61)	10 (9.43)	13 (6,88)	4.99 (1.1–22.8)	0.038
Relative^$^	2 (66.7)	9 (90.0)	11 (84.6)		
Medical delegate	1 (33.3)	1 (10.0)	2 (15.4)		
**Personal motivating**	21 (25.3)	37 (34.91)	58 (30.7)	2.64 (1.01–6.92)	0.048
Drug-taking practice	6 (28.6)	9 (23.3)	15 (25.4)		
Finance cost reduction	1 (4.8)	1 (2.7)	3 (5.1)		
Prevention	1 (4.8)	4 (10.8)	5 (8.5)		
Renewal treatment or Resumption	6 (28.6)	8 (21.6)	14 (23.7)		
Family medication	1 (4.8)	1 (2.7)	2 (3.4)		
Recidivism	6 (28.6)	14 (37.8)	20 (33.9)		

Data are number and/or proportion (%)

^*$*^, Friends, neighbors or parents.

### Dispensing practices in pharmacies

The role of pharmacy auxiliaries in antibiotics dispensing is predominant as 78.4% of participants received their antibiotic from an auxiliary, whereas only 21.6% of customers received their from a pharmacist (Table 4). Almost all of the customers (94.8%) were given explanations on dosage and precautions for use by one prescriber and/or dispenser. Participants with a prescription were seven times more likely to receive an explanation about antibiotics use than those who self-medicating with antibiotics (OR = 7.37, 95%CI:2.13–25.43, p = 0.002).

**Table 4:** Dispensing practices in the pharmacies.

	Self-medication			Prescription			Total
	Female	Male	Total	Female	Male	Total	
**Dispensing**	83	106	189	110	103	213	402
Pharmacy auxiliaries	60 (72.3)	81 (76.4)	141 (74,6)	93 (84.6)	81 (78.6)	174 (81,69)	315 (78.4)
Pharmacists	23 (27.7)	25 (23.6)	48 (25,4)	17 (15.4)	22 (21.4)	39 (18,31)	87 (21.6)
**Explanation on instructions of use and precautions**	74 (89,2)	97 (91;5)	171 (90,48)	109	101	210 (98,59)	381 (94.8)
Practitioners	0 (0.0)	1 (1.0)	1 (0,6)	11 (10.1)	7 (6.9)	18 (8.6)	19 (5.0)
Practitioners & Pharmacy auxiliaries	0 (0.0)	2 (2.1)	2 (1,2)	48 (44.0)	38 (37.6)	86 (40.9)	88 (23.1)
Practionners & Pharmacysts	0 (0.0)	0 (0.00)	0 (0,0)	17 (15.6)	19 (18. 8)	36 (17.1)	36 (9.4)
Pharmacysts	22 (29.7)	24 (24.7)	46 (26,9)	2 (1.8)	3 (3.0)	5 (2.4)	51 (13.4)
Pharmcysts & Nurses	0 (0.0)	0 (0.0)	0 (0,0)	0 (0.0)	1 (1.0)	1 (0.5)	1 (0.3)
Nurses	1 (1.3)	0 (0.0)	1 (0,6)	2 (1.8)	0 (0.0)	2 (0.9)	3 (0.8)
Nurses & Pharmacy auxiliaries	5 (6.8)	4 (4.1)	9 (5,3)	15 (13.8)	21 (20.8)	36 (17.1)	45 (11.8)
Pharmacy auxiliaries	46 (62.2)	66 (68.0)	112 (65,50)	14 (12.8)	12 (11.9)	26 (12.4)	138 (36.2)

Data are number and/or proportion (%).

Over all, the main sources of information on antibiotics use were pharmacy auxiliaries (274/381, 71.9%), whereas practitioners and pharmacists accounted for 37.5% (143/381) and 23.1% (88/381) respectively. Among patients with a prescription who received explanations, 66.7% (140/210) reported having received informations about using antibiotics from their practitioner. Practitioners account for the 73% of prescribers and provide less explanations about the antibiotics use than the pharmacy auxiliaries (66.7% *vs* 71,4%, OR = 0.205, 95%CI:0.09–0.46, p<0.0001), but more than pharmacists (66,7% *vs* 20.0%, OR = 3.692; 95%CI:1.437–9.25, p = 0.005). In self-medication, pharmacy auxiliaries remain the main persons who give explanation on antibiotics use compared to pharmacists (72.5% *vs* 26.9%).

### Medication and associated factors by age groups

The mean age of the adult consumers of antibiotics (ACA) was 36.1 [min:15, max:78] years and the male/female sex ratio was 0.8. Among ACA, males (38 years) tended to be older than females (mean 34.6) (p = 0.0049). Overall, there was no significant difference in the mean age between ACA between prescription and self-medication (Table 5), however self-medication was significantly higher among patients aged 41 years and older compared to participants of 36–40 years old (p = 0.004) and to patients 21–25 years old (p = 0.015). Self-medication was, on average, twice as common in adults under 41 years old practiced (OR = 1.97, 95%CI:1.11–3.49, p = 0.021).

**Table 5:** Multivariate analysis of factors associated with type of medication according to antibiotic target groups.

Antibiotics target groups	Self-medication	Prescription	Total	Univariate analysis^#^		Multivariate analysis^#^	
				OR (95%CI)	p	OR (95%CI)	p
**ADULTS**^**$**^							
**Gender**	93 (38,1)	151 (61,9)	244				
Female*	38 (28.2)	97 (71.8)	135 (55.3)	Reference			
Male	55 (50.5)	54 (49.5)	109 (44.7)	2.60 (1.53–4.42)	<0.0001	2.22 (1.17–4.18)	0.014
**Mean age (Sd), years**	38.2 (13.0)	34,8 (11,1)	36.1 (11.9)	/	0.034		
< = 40*	60 (64.5)	118 (78.1)	178 (72.9)	1.97 (1.11–3.49)	0.021	2.18 (1.07–4.46)	0.032
>48	33 (35.5)	33 (21.9)	66 (27.1)	Reference			
**Educational level**^**£**^	69 (39.0)	108 (61.0)	177	2.07 (1.10–4.00)	0.031		
Out-of-school & primary*	8 (11.6)	17 (15.7)	25 (14.1)	Reference			
Secondary	28 (40.6)	58 (53.7)	86 (48.6)	1.02 (0.94–2.66)	0.958		
University	33 (47.8)	33 (30,6)	66 (37.3)	2.07 (1.10–4.00)	0,031	2.14 (1.11–4.09)	0.022
**CHILDS**							
Gender	96 (60.8)	62 (39.2)	158				
Boys	49 (61.2)	31 (38.8)	80 (50.6)	Reference			
Girls	47 (60.3)	31 (39.7)	78 (49.4)	1.04 (0.55–1.97)	0.898		
Mean age (Sd), years	2.73 (2.6)	2.59 (2.4)	2.7 (2.5)	/	0.801		

Data are number and/or proportion (%), unless otherwise indicated

^$^, People over 15 years

*, Variables used as reference

£, Analysis on the impact of these variables was done only among people who came to buy their own antibiotics

^#^, Comparison made between self-medication and prescription groups for the variable indicated.

Around 72% (177/244) of ACA have purchased their own drugs and 61.89% (151/244) of antibiotics were prescriptions (Table 5). Women were more likely than men to have a prescription for their antibiotics (78.1% *vs* 49.5%, OR = 2.60, 95%CI:1.53–4.42, p<0.0001). Having sparse information on education level and occupation of adults to whom the drug was administered, analysis on the impact of these two variables was done only among people who came to buy their own antibiotics. Non-prescription use was positively associated with higher education compared to secondary education (OR = 2.07, 95%CI:1.10–4.00, p = 0.031) or compared to no/primary/secondary education (OR = 2.08, 95%CI:1.11–3.89, p = 0.021). After multivariate analysis, male (OR = 2.32, 95%CI:1.24–4.34, p = 0.009) and higher education (OR = 2.05, 95%CI:1.08–3.89, p = 0.027) remained significantly associated with self-medication (Table 5).

Overall, 39.3% of the antibiotics purchased were for children under 15 years of age with a sex ratio male/female of 1.02 (Table 5). The mean age of children who received antibiotics was 2.67 (Sd = 2.54) years, and and girls (mean = 3.10 years, Sd = 2.78) were significantly older than boys (mean = 2.27 years, Sd = 2.24) (p = 0.035). Children aged 1 to 5 years accounted for more than 60% of dispensed antibiotics (Fig 2B). No significant differences were observed with regard to mean age or gender of children and type of medication. Around 61% (96/158) of children were self-medicated by their parents, 62.03% for women and 59.48% for men (p = 0.435). There was a non-significant trend for women with low scores for knowledge about the role of antibiotics to give their children antibiotics without a prescription compared to other women (p = 0.08). No association between education levels, professional activities or age groups of parents and types of children medication was found.

## Discussion

As antibiotics have been touted in certain communities as able to "treating everything" and their access is unregulated in developing countries, the abusive use of antibiotics is generally recognized [10–13,32] and bacterial resistance to antibiotics is frequently reported in these countries [1, 19, 24–26,31–34]. But the practice of how we produce, prescribe and use antibiotics remains unknown in many African countries. To our knowledge, this study is the first to investigate the knowledge, attitudes, and behaviors of adults concerning antibiotics and how these medications are dispensed (prescription or self-medication) and used by the Cameroonian population. This study is also among the few studies conducted in the non-health care profession in Africa to date [25,35].

For 3 months, we interviewed 402 of 1,192 customers attending pharmacies and who bought antibiotics. Children aged 0–14 account for 39.3% of antibiotic users; the largest proportion is due to children aged 1 to 5 years.

Our findings showed fairly poor knowledge about the role of antibiotics. More than 87% of respondents (≥ 15 years old) were not aware that antibiotics drugs are not effective against all microbes and only 43.7% of respondents believed that antibiotics induce bacteria death. Over 10 percent (11.8%) of the participants also believed antibiotics also believed antibiotics could be used to treat viral infections. It has been previously reported in community-based studies in developing countries that many adults (≥18 years old) do not understand the differences between bacteria and viruses and believe that antibiotics act against both. For example, less than 40% of participants believed that antibiotics are effective against bacteria, while 6.9% and 48.1% incorrectly believed that antibiotics are effective against viruses in two studies [25, 5]. The misunderstanding of antibiotics as potentially effective as antitussives, painkillers or antipyretics has been reported in this study and others [35, 36]. The level of knowledge among customers seeking to buy antibiotics is well below that found among health professionals or drug sellers [34, 37, 38]. Similar to previous survey [25], having a professional activity in the private or state sector or having a higher level of education was significantly associated with a fairly good knowledgeable about antibiotics. Therefore, educational campaigns should be focus on pupils/students, retired/unemployed and in informal sector.

In the matter of knowledge of antibiotics’ side effects or risks, although overall respondents stated that antimicrobial drugs can cause adverse reactions, only 58% were able to name one side effect, among which allergic reaction was the most often reported (52.8%). Insufficient knowledge about the use of antibiotics could explain their inappropriate use and, even possibly serious and adverse drug effects due to their abuse.

The practice of self-medication is worldwide, more than 30% and up to 75% among adults in some sub-Saharan Africa countries [10–13, 15, 25,26] and between 5–50% in industrialized countries [1, 4, 5, 35]. These high percentages are comparable to that reported in our study with 38% of adults using antibiotics directly from pharmacies and without any physician’s prescription. Although policies exist to regulate antibiotic use, these results highlight that access to antibiotics among the population of Cameroon remains uncontrolled and suggest that enforcement, and education on antibiotic use and antimicrobial resistance are insufficient or lacking in our context. Male sex, age under 35 years old and higher education were reported as major factor associated with higher risk of self-medication. In previous population surveys, females were more likely than males to take antibiotics only when prescribed, and the increased risk of self-medication were also significantly associated with middle aged respondents of 40–59 years, lower income and higher level of education [35, 36, 38, 39–41]. The differences in the prescription between the two genders may be explained in part by more available time of female than male since women have more informal or unemployed activity than men who have a stable activity.

According to studies conducted in Europe, United States and in some African areas, the rate of pediatric self-medication is very high, up than 50% and reaching 60–90% [7–9, 40, 42– 44]. Our study is consistent with these previous studies, with 60.8% of parents who practiced self-medication for their children. Contrary to a study conducted in Yemen [42], the present study and others did not find child gender to be associated with self-medication. One of the explanations given by the authors was that many families in Yemen prefer male children than females, so they seek medical advice for boys early without trying antibiotics unprescribed. The parents or relatives did not use self-medication with antibiotics for children below 12 months without a medical consultation. Our survey of pharmacists and parents showed that, both are afraid and also refuse to give medicines, including antibiotics to very young children since the risks associated with the antibiotics use are even higher in this group of patients. Previous studies have shown over 70% of mothers were more likely to give their children antibiotics without a prescription and the mothers with a higher score for knowledge of the antibiotics use and their hazards were less likely to give their children antibiotics without a prescription [9, 43, 45]. In our study, a non-significant trend was also found among females between low score for knowledge of antibiotics and giving children antibiotics without a prescription (p = 0.08). They are two hypotheses that could explain this low difference: first, the small sample size of 79 (62%) mothers who bought antibiotics for their children without a prescription may not have had the power to detect a significant different, and second, some women may ask husbands to pick up antibiotics from a pharmacy on their way home from work. This could lead to observer bias in this prevalence in our study (62.03% *vs* 59.49% for father) when in fact; it was mothers who are overwhelmingly responsible for self-medication for children rather than fathers.

Many factors may explain the variation in the prevalence of self-medication among different studies, including the study area (hospital, pharmacy, and community), the healthcare services and the culture. Main reasons given for the practice of self-medication in developing countries include proximity of pharmacies to their residence or long distance to healthcare facility, lack of money, ignorance, mild/minor illness, poor attitude of health workers (rude, corrupt, dirty) re-treatment of similar illness and lack of health personnel [46, 47]. Moreover, in our context, we observed in other studies that the majority of patients referred to a health facility only when they cannot control the symptoms of the disease or when the life-threatening prognosis is often at stake.

Self-medication can be justified when it is given by a health worker because, it minimizes the risks and dangers associated with it, and it’s a responsible self-medication [48], and a component of self- care recognized by WHO especially in area where personnel are insufficient [49]. In Cameroon, the patient-physician ratio is less than 1.5 physicians per 1,000 patients and patients report difficulties in accessing routine or ongoing care preferring instead to go directly to pharmacies in order to reduce hospital expenses [50]. In this context, it is commonly accepted that self-medication also has advantages for healthcare systems as it reduces pressure on medical services, facilitates better use of clinical skills of pharmacists and the paramedics and who also prescribe medications, increases access to medication helping patient to take care of minor ailments, and may contribute to reducing prescribed drug costs and time spent in accessing health care [49, 51]. However, free access to antibiotics and other medicines may encourage patients to believe that there is a pharmacological treatment for every ailment leading to the abuse of these drugs. Abuse of prescription drugs exists as type of malpractice which can delay or mask the diagnosis of serious illnesses, increase the risk of adverse drug reactions, or drug-drug interactions, result in inadequate dosing or polypharmacy [46, 52–55]. Unfortunately all these contribute to the emergence and spread of antibiotic drug resistance reported in many countries [2, 19–22]. The results clearly show that the population needs more education on the use of antibiotics. The effectiveness of information, education and communication to ensure the appropriate use of antimicrobials among public could be achieved by internet and social media use within the public health communication strategy. Recent studies have shown that internet and social media are widely used for antibiotic-related information seeking in the Italian population [56, 57].

Requiring a prescription from a medical profession is one formal solution; however in our context, non-physicians may also prescribe medications. This explains the unexpectedly high prevalence (26.84%) of reported paramedical prescriptions (nurses, midwives and laboratory technicians). In this context, having a prescription does not guarantee that a correct diagnosis has been made by a qualified health care provider [26,58]. Interestingly, 96% of respondents who come to buy antibiotics agreed with the statement that antibiotics should be access-controlled drugs prescribed by a physician; this prevalence is higher than that reported in previous studies, 48% and 62% [25, 59]. Similarly, dispensing drugs is a pharmaceutical act, but more than 78% of dispensers were the pharmacy assistants. This is supported by other studies showing that prescriber is not a physician [37] and around 70% of prescriptions are dispensed by pharmacy auxiliaries [13, 37]. This can be explained by the fact that pharmacists have other activities outside the pharmacy and that the profession no longer requires their permanent presence for the extemporaneous preparation of medications.

Despite some limitations, this study presents several strengths, including health district-wide scale, and collection of many variables. To our knowledge, this is the first study from Cameroun linking antibiotic purchase and dispensing practices in the pharmacies at health district-wide scale to customers use, knowledge and perceptions of antibiotics. Most of the few studies carried out (China, Democratic Republic of Congo, India, USA, Jordan and European countries) have a relatively low sample size and most of them concern medical students [5, 34, 60–68]. In this study, we selected all the 13 pharmacies located in the 4^th^ District of Douala to improve representativeness of our convenience sample. Two pharmacies were excluded for low patients flow (3–5 people per day) due to the limited time available to the Pharmacy student for his thesis and four pharmacies were excluded for the repeated absence of the pharmacist to obtain the approval. Moreover, contrary to many studies, rather than a self-administered questionnaire, patients were interviewed directly. In addition, the questionnaire explicitly assessed whether the antibiotics were prescribed or recommended by a physician and whether the antibiotics were bought over the counter without a prescription. No information was available on the proportion of privately insured parents or relatives among all participants. Although our findings cannot be applied to all regions of Cameroon, this work has important implications for providing additional insight the preventive options of antibiotics missuses.

## Conclusions

Misuse, little "practical knowledge" and high self-medication confirm the unsatisfactory prescription and dispensing practices of the antibiotics in Cameron, like many other developing countries. Our findings suggest an antibiotic misuse among children through high self-medication by their parents and for themselves, and mothers than men. Similar studies should be repeated in other health districts in order to identify the most appropriate strategies for each local to improve the quality of health education among community members and of antibiotic use guidelines with the ultimate goal of controlling misuse of antibiotic and subsequent antibiotic resistance. These results highlight the important of the development and implementation of appropriate guidelines for the responsible use of antibiotics for health care providers and health education targeting community members themselves.

## Abbreviations

AAP: Attitude and Practice
ACA: Adult consumers of antibiotics
CEIQ: Closed-Ended Interview Questions
FMPS: Faculty of Medicine and Pharmaceutical Sciences
INN: International Nonproprietary Name
IQ25-IQ-75: Second and fourth InterQuartile (IQ) above the median
IRB/UD: Institutional Review Board of the University of Douala
MDRBs: Multi-Drug Resistant Bacteria
OEIQ: Open-Ended Interview Questions
OTC: Over-The-Counter
WHO: World Health Organization
